# Functional *EPAS1*/*HIF2A* missense variant is associated with hematocrit in Andean highlanders

**DOI:** 10.1126/sciadv.adj5661

**Published:** 2024-02-09

**Authors:** Elijah S. Lawrence, Wanjun Gu, Ryan J. Bohlender, Cecilia Anza-Ramirez, Amy M. Cole, James J. Yu, Hao Hu, Erica C. Heinrich, Katie A. O’Brien, Carlos A. Vasquez, Quinn T. Cowan, Patrick T. Bruck, Kysha Mercader, Mona Alotaibi, Tao Long, James E. Hall, Esteban A. Moya, Marco A. Bauk, Jennifer J. Reeves, Mitchell C. Kong, Rany M. Salem, Gustavo Vizcardo-Galindo, Jose-Luis Macarlupu, Rómulo Figueroa-Mujíca, Daniela Bermudez, Noemi Corante, Eduardo Gaio, Keolu P. Fox, Veikko Salomaa, Aki S. Havulinna, Andrew J. Murray, Atul Malhotra, Frank L. Powel, Mohit Jain, Alexis C. Komor, Gianpiero L. Cavalleri, Chad D. Huff, Francisco C. Villafuerte, Tatum S. Simonson

**Affiliations:** ^1^ Division of Pulmonary, Critical Care, Sleep Medicine, and Physiology, Department of Medicine, University of California, San Diego, La Jolla, CA, USA.; ^2^ Department of Epidemiology, University of Texas MD Anderson Cancer Center, Houston, TX, USA.; ^3^ Laboratorio de Fisiología Comparada/Fisiología de del Transporte de Oxígeno-LID, Departamento de Ciencias Biológicas y Fisiológicas, Facultad de Ciencias y Filosofía, Universidad Peruana Cayetano Heredia, Lima, Perú.; ^4^ School of Pharmacy and Biomolecular Sciences, Royal College of Surgeons in Ireland, Dublin, Ireland.; ^5^ Division of Biomedical Sciences, School of Medicine, University of California, Riverside, Riverside, CA, USA.; ^6^ Department of Physiology, Development and Neuroscience, University of Cambridge, Cambridge CB2 3EG, UK.; ^7^ Department of Chemistry and Biochemistry, University of California, San Diego, La Jolla, CA, USA.; ^8^ Department of Anthropology and Global Health, University of California, San Diego, La Jolla, CA, USA.; ^9^ Department of Medicine and Pharmacology, University of California, San Diego, La Jolla, CA, USA.; ^10^ Sapient Bioanalytics, LLC, San Diego, CA, USA.; ^11^ Department of Bioengineering, University of California, San Diego, La Jolla, CA, USA.; ^12^ Herbert Wertheim School of Public Health and Longevity Sciences, University of California, San Diego, La Jolla, CA, USA.; ^13^ Laboratório de Fisiologia Respiratória, Faculdade de Medicina, Universidade de Brasília, Brasília, Brazil.; ^14^ Department of Public Health and Welfare, Finnish Institute for Health and Welfare, Helsinki, Finland.; ^15^ Institute for Molecular Medicine Finland (FIMM-HiLIFE), Helsinki, Finland.

## Abstract

Hypoxia-inducible factor pathway genes are linked to adaptation in both human and nonhuman highland species. *EPAS1*, a notable target of hypoxia adaptation, is associated with relatively lower hemoglobin concentration in Tibetans. We provide evidence for an association between an adaptive *EPAS1* variant (rs570553380) and the same phenotype of relatively low hematocrit in Andean highlanders. This Andean-specific missense variant is present at a modest frequency in Andeans and absent in other human populations and vertebrate species except the coelacanth. CRISPR-base-edited human cells with this variant exhibit shifts in hypoxia-regulated gene expression, while metabolomic analyses reveal both genotype and phenotype associations and validation in a lowland population. Although this genocopy of relatively lower hematocrit in Andean highlanders parallels well-replicated findings in Tibetans, it likely involves distinct pathway responses based on a protein-coding versus noncoding variants, respectively. These findings illuminate how unique variants at *EPAS1* contribute to the same phenotype in Tibetans and a subset of Andean highlanders despite distinct evolutionary trajectories.

## Teaser

Variation in hematocrit is mediated by different variants and mechanisms involving the same gene (*EPAS1*) in Tibetans and Andeans.

## INTRODUCTION

High-altitude populations exhibit phenotypes that reflect several hundred generations of genetic adaptations to environmental stress. Many Tibetan and Andean groups have distinct physiological traits that mitigate the decreased availability of oxygen (O2) at high altitude via augmented O2 transport and utilization as well as protection from reproductive and neonatal complications (*1*, *2*). Only a few of these traits have been linked to genetic factors, including relatively lower hemoglobin concentration ([Hb]) (*3*–*5*) and altered metabolic function within Tibetans (*6*, *7*) and increased uterine artery diameter, birth weight (*8*), and exercise capacity within Andeans (*9*). However, the extent of adaptation involving the same genetic regions across continental highland populations remains an ongoing area of research (*10*, *11*).

Numerous genomic selection studies have implicated a wide range of candidate genes as targets for human high-altitude adaptation (*3*–*5*, *9*, *12*–*28*). Many of the strongest signals of natural selection emanate from genes within the hypoxia-inducible factor (HIF) pathway, a master transcriptional regulator of cellular responses to low O2 (*29*). *Endothelial PAS Domain Protein 1* (*EPAS1*), which encodes the alpha subunit of HIF-2 (*29*), is one of the top selection targets identified in humans (*1*, *30*) and other highland species (*31*, *32*). In addition, variants within *EPAS1* have been associated with relatively lower [Hb] in Tibetan groups, which are linked, indirectly or directly, to an adaptive phenotype [e.g., protection from excessive erythrocytosis (EE)] (*3*, *5*). Although Tibetans demonstrate lower average [Hb] than Andeans, Andean highlanders exhibit substantial intrapopulation variation in this phenotype (*1*), with values ranging from sea level to excessively high levels (*33*). While many of the same candidate gene regions studied in Tibetans have been identified as potential targets of selection in Andeans (*12*), including *EPAS1* (*10*, *25*), adaptations upon the same genes and associated phenotypes have not been found.

Here, we examine a previously identified *EPAS1* missense variant (rs570553380, A>G, p.[His194Arg]) (*10*, *25*) within a cohort of Andean highlanders (Cerro de Pasco, Peru, 4340 m). Through genotype-phenotype analyses and in vitro functional assessments, we show evidence of genotypic-phenotypic, cross-continental convergence between Andean and Tibetan highland groups. We demonstrate that lower hematocrit and altered transcriptional activity is associated with a putatively adaptive, Andean-specific missense *EPAS1* variant. In addition, we identify unique metabolomic signatures associated with this *EPAS1* allele and hematocrit.

## RESULTS

### Positive selection of *EPAS1* variant rs570553380 in Andean highlanders

**Fig. 1.**
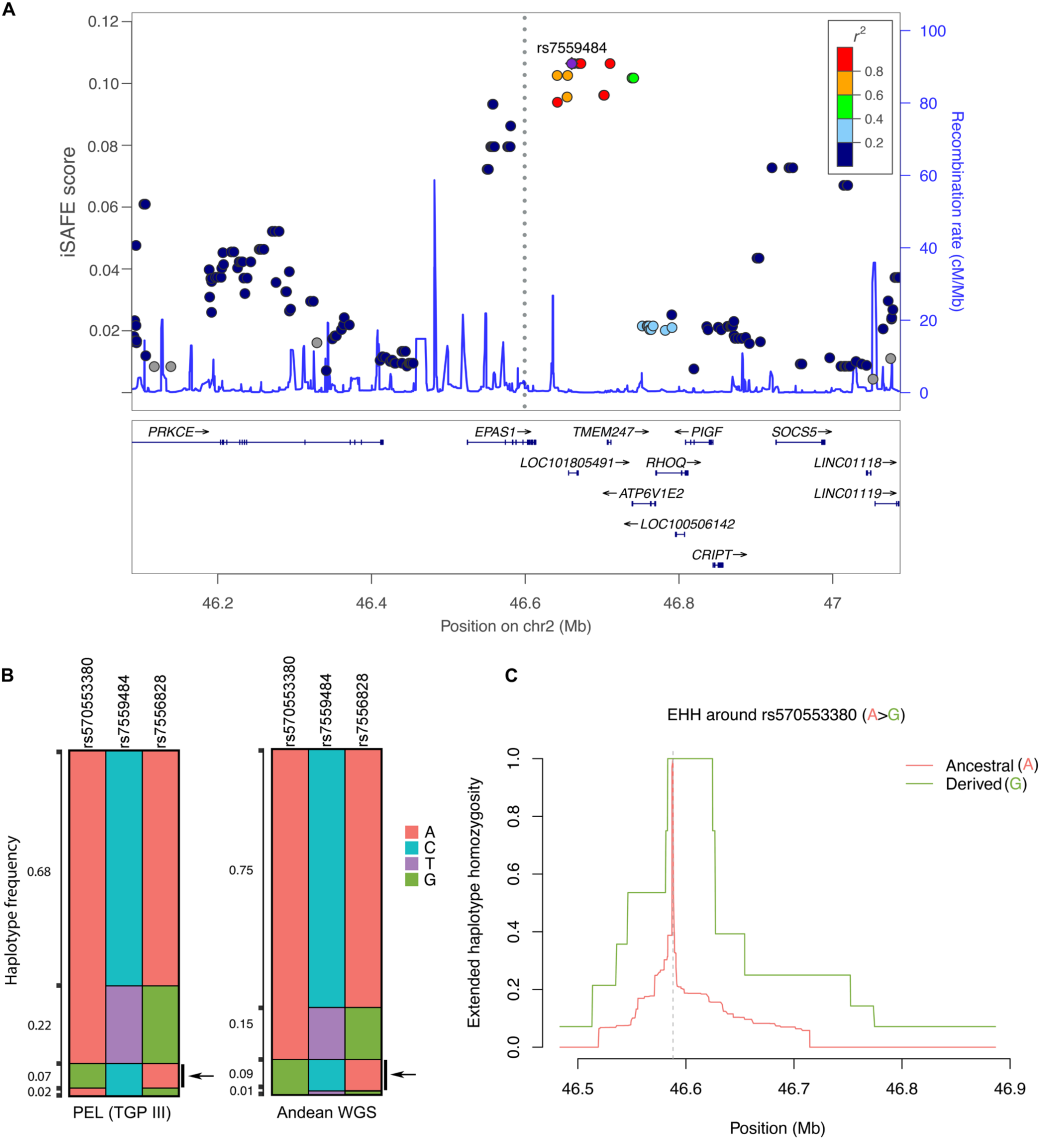
*EPAS1* variant under positive selection in Quechua Andean highlanders. (**A**) iSAFE results from the 1-Mb window surrounding rs570553380. Regional plots of iSAFE scores (*34*) generated from the *EPAS1* locus in the Andean whole genomes and visualized using Locuszoom. The left *y* axis shows the iSAFE score ranging from 0 to 1. Markers with iSAFE scores equal to or greater than 0.10 are considered favored by natural selection. The right *y* axis denotes the recombination rate in hundred crossovers per million base pairs (cM/Mb). The gray dotted line indicates the position of *EPAS1* rs570553380 variant (unscored), and the purple diamond denotes the marker in the region with the highest iSAFE score (rs7559484). SNVs in the region are denoted in circles, color-coded based on linkage disequilibrium (LD) with the top iSAFE marker. (**B**) Haplotype-frequency plot of haplotypes in 1000 Genomes PEL subpopulation (*n* = 170 haplotypes) and the Andean whole genomes (WGS) (*n* = 80 haplotypes) containing rs570553380 and top iSAFE markers (rs7559484 and rs7556828). The black arrow indicates the haplotypes carrying the putatively adaptive “G” allele of rs570553380, in which the “C” and “A” alleles of respective iSAFE markers rs7559484 and rs7556828 are nearly always present (D′ > 0.99). (**C**) EHH plot around rs570553380 (A>G) in the 40 Quechuan Andean whole genomes.

We sequenced the whole genomes of 40 Quechua Andean highlanders (figs. S1 and S2) and examined variants within a single-megabase region surrounding a protein-coding site at rs570553380 using integrated Selection of Allele Favored by Evolution (iSAFE) (*34*) (Fig. 1A). We observed a signature of selection at this locus as similarly reported in a study of early-stage selection in Colla highlanders (*25*). The top iSAFE signal localized to a set of intergenic variants (rs7559484, T>C and rs7556828, G>A) approximately 70 kb upstream of rs570553380, which could not be tested directly by iSAFE because of its modest allele frequency. Haplotype analysis of rs7559484, rs7556828, and rs570553380 in the Andean whole genomes (*n* = 80 haplotypes) and 1000 Genomes Peruvian (PEL) subpopulation (*n* = 170 haplotypes) showed the putatively adaptive allele of rs570553380 (G) is inherited with derived alleles rs7559484 (C) and rs7556828 (A) (Fig. 1B) (D′ > 0.99). Furthermore, these derived alleles are enriched in Andean highlanders (both 83.8%) compared to the global average (45 and 44%, respectively), suggesting that early-stage selection of rs570553380 may have contributed to an increase in their frequencies. Extended haplotype homozygosity (EHH) (Fig. 1C) and integrated haplotype score (iHS) analyses of rs570553380 in Andeans highlight a signal of natural selection at rs570553380 (G), in which haplotypes with the derived allele (G) were significantly longer than those with the ancestral allele (A) [iHS = −2.43, *P* < 0.02; |iHS| > 2 indicates strong selection (*35*)]. In contrast, EHH and iHS analyses of rs7559484 and rs7556828 demonstrated no significant differences in haplotype length (fig. S3), suggesting that selection at the *EPAS1* locus in Andeans may be attributed to rs570553380.

Although the allele frequency of rs570553380 (A>G) is modest among Andean highlanders [Quechua: 10% in this study; Quechua: 9% (*10*); Colla: 32% (*25*)], rs570553380 (G) is nearly absent in publicly available genome data with a global frequency of only 0.20% [attributed solely to the Peruvian subpopulation (PEL) = 7.1%] (1000 Genomes, Phase III). The estimated age of rs570553380 is ~9845 years (Genealogical Estimation of Variant Age (GEVA), based on 1000 Genomes data) and ~13,027 years (based off calculations of haplotype heterozygosity in the 40 Andean whole genomes) (table S1). These estimates align approximately with the first human habitation of the Andes (*1*, *36*) and suggest this variant may have originated within early settlers of this region. The age estimates of this variant are consistent with its modest allele frequency in Andeans and early-stage selection at this locus.

### Positively selected *EPAS1* allele is associated with relatively lower hematocrit in Andeans

Putatively adaptive variants in *EPAS1* are associated with significantly lower [Hb] within Tibetan individuals (*3*, *5*). Hematocrit (Hct), the percent volume of red blood cells in whole blood is, on average, higher in Andeans than Tibetans (*1*) and proportional to [Hb] (Hct ≈ 3*[Hb], [Hb] in grams per deciliter). EE, an overproduction of red blood cells and hallmark of chronic mountain sickness (CMS), is more prevalent in Andean than Tibetan men and associated with pulmonary hypertension, myocardial infarction, and cor pulmonale (*33*).

**Fig. 2.**
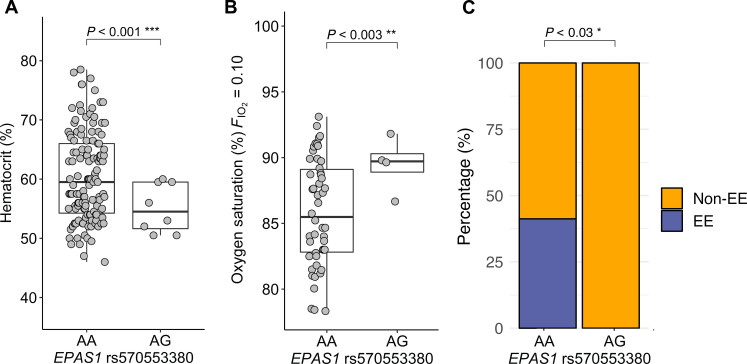
Positively selected *EPAS1* variant is associated with lower hematocrit, higher oxygen saturation, and lower incidence of EE within Andean males. In Andean males, the *EPAS1* missense variant rs570553380 (A>G, p.[His194Arg]) is associated with (**A**) hematocrit (%) (*n* = 139), (**B**) oxygen saturation (%SpO2) at $F_{IO_{2}}$ = 0.10 [subset of the 139 Andeans, *n* = 55; data from (*40*)], and (**C**) incidence of EE (*n* = 139). EE criteria: Hct ≥ 63% in men ([Hb] ≥ 21 g/dl) and ≥ 57% in women ([Hb] ≥ 19 g/dl) (*63*) (see fig. S4 for females). *P < 0.05, **P < 0.01, and ***P < 0.001. NS, not significant.

We tested whether rs570553380 (A>G, p.[His194Arg]) was associated with Hct in an expanded cohort of Andean highlanders (*n* = 139 males, 49 females) (table S2) and validated via a permutation test (fig. S5). Given women have a lower prevalence of EE before menopause (*33*, *37*, *38*) and estrogen has a protective effect on EE (*39*), analyses were stratified by sex. Males with one copy of the putatively adaptive allele “G” (Arg) demonstrated a significantly lower Hct (*n* = 139) (*P* < 0.001) (Fig. 2A), higher O2 saturation at 10% inspired O2 ($F_{IO_{2}}$ = 0.10) [*n* = 55, subset of 139 Andean males (*40*)] (*P* < 0.003) (Fig. 2B), and lower incidence of EE (*n* = 139) (*P* < 0.03) (Fig. 2C) compared to individuals with the ancestral allele. However, these relationships were not significant in females (fig. S4), most likely due to a smaller sample size of females and well-described differences in Hct and EE with respect to sex and age (before and after menopause) (*33*, *39*, *41*) (table S2). Primary and/or secondary effects of this variant on these phenotypes and interplay with other O2 transport components, as well as expanded studies in women, are necessary to understand the underlying physiological mechanisms.

### *EPAS1* site is highly conserved and adaptive variant attenuates expression of HIF target genes

**Fig. 3.**
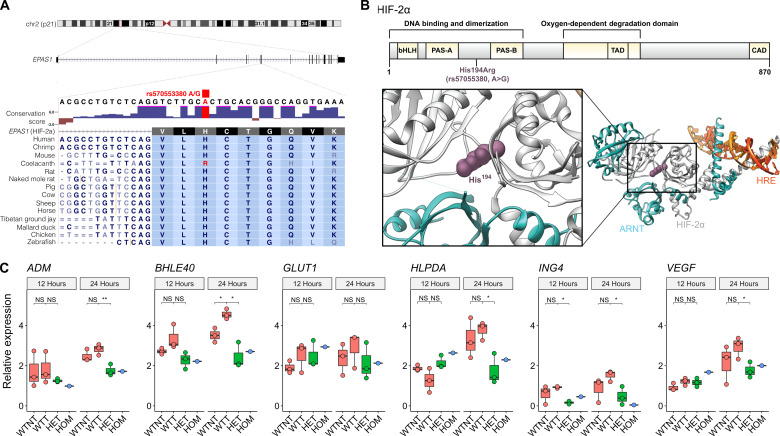
*EPAS1* variant is highly conserved and results in altered expression of HIF-2α targets under hypoxia. (**A**) Conservation of rs570553380 (A>G) in a subset of 100 Species Vertebrate Multiz Alignment & Conservation (fig. S6). Conservation scores (phyloP) of nucleotides across 100 vertebrates showed significant conservation at rs570553380 [−log(*P*) = 6.02; positive scores indicate slower evolutionary change while negative scores indicate accelerated evolution, greater than expected due to neutral drift (*42*)]. (**B**) Location of rs570553380 within the ribbon diagram and crystal structure of HIF-2α (white) as bound to aryl hydrocarbon receptor nuclear translocator (ARNT) (teal) and the hypoxia response element (HRE, orange) (Protein Data Bank ID: 4ZPK). His^194^ (purple), shown in sphere representation, extends into one of the protein-protein interfaces between HIF-2α and ARNT. (**C**) Relative expression of HIF-2α target genes *Adrenomedullin* (*ADM*), *Basic Helix-loop-helix Family Member e40* (*BHLE40*), *Solute Carrier Family 2 Member 1 (GLUT1*), *Hypoxia Inducible Lipid Droplet Associated* (*HILPDA*), *Inhibitor of Growth Family Member 4* (*ING4*), and *Vascular Endothelial Growth Factor A* (*VEGF*) (table S5) in HEK293T cells nontransfected (WTNT, *n* = 3) and transfected wild-type (WTT, *n* = 3), heterozygous (HET, *n* = 3), and homozygous (HOM, *n* = 1) for rs570553380 (A>G) exposed to hypoxia (1% O2) relative to normoxia (21% O2) for 12 and 24 hours. Gene expression was determined by real-time reverse transcription quantitative polymerase chain reaction (RT-qPCR). Two-way analysis of variance (ANOVA) shows a significant effect of rs570553380 on gene expression of *ADM*, *BHLE40*, *HILPDA*, *ING4*, and *VEGF* after 24-hour exposure to hypoxia, as well as a transfection effect on *BHLE40* gene expression. The post hoc generalized linear model shows that increased expression of *ADM*, *BHLE40*, *HILPDA*, *ING4*, and *VEGF* from 12- to 24-hour exposure to hypoxia is significantly lower in HET compared to WTT. *P < 0.05 and **P < 0.01.

Analysis via phyloP (conservation score) (*42*) indicates significantly greater conservation of rs570553380 (A>G) than expected due to neutral drift [−log(*P*) = 6.02] (Fig. 3A). A comparison of rs570553380 (A>G, p.[His194Arg]) across vertebrates at the amino acid level revealed the wild-type histidine residue (His^194^) as highly conserved across species except the coelacanth, *Latimeria chalumnae*, a lobe-fined fish found in the depths of the West Indian Ocean. During the past 100 million years, coelacanths have migrated from shallow-brackish to deeper marine waters, during which extreme selective pressures, such as hypoxia, may have acted (*43*). Although humans and coelacanths diverged more than 400 million years ago (*44*), this species of lobe-finned fish has the same arginine residue as coded by rs570553380 in Andean highlanders (fig. S6), suggesting potential convergence at this locus.

In silico analysis of rs570553380 (A>G, p.[His194Arg]) (Fig. 3B) predicted the Arg residue as destabilizing and deleterious in humans (table S3) (*45*–*47*), suggesting the *EPAS1* variant may alter HIF-2α structure, stability, and/or transcriptional activity. To assess potential transcriptional effects of rs570553380, we introduced this variant into human endothelial kidney (HEK) 293T cells using CRISPR-Cas9 ABE base editing (*48*) (fig. S7). Potential off-target mutations from transfection and clonal expansion were assessed via whole-exome sequencing (WES), in which no increase in exome-wide A•T to G•C mutations was observed (fig. S8 and table S4). To evaluate the transcriptional effects of this *EPAS1* variant in response to hypoxia, isogenic wild-type, heterozygous, and homozygous clones were cultured under conditions of normoxia (21% O2) and hypoxia (1% O2) for 12 and 24 hours. The selection of these conditions were based on previous literature in which these O2 tensions and time points were implemented to study HIF activity in HEK293T cells (*49*–*52*).

Cells with one copy of the putatively adaptive G (Arg) allele demonstrated lower expression of numerous canonical HIF-2 target genes (Fig. 3C) despite showing HIF-2α levels similar to those of wild-type cells under hypoxia (fig. S9). In particular, expression of *ADM*, *BHLE40*, *HILPDA*, *ING4*, and *VEGF* (table S5) was significantly decreased in cells heterozygous for the *EPAS1* variant relative to wild-type cells from 12- to 24-hour hypoxia exposure relative to normoxia (Fig. 3C). Wild-type cells exhibited lower levels of HIF-2α than cells heterozygous for SNV rs570553380 in normoxia, suggesting potential compensatory changes in response to altered HIF activity (fig. S9). The observed down-regulation of canonical HIF targets in cells with the Andean-specific missense variant parallels the loss-of-function phenotype similarly observed in studies of this variant (*10*) and Tibetan-specific noncoding variants in *EPAS1* (*11*, *53*, *54*). While these findings provide insight into altered transcriptional activity as a result of this missense variant in vitro, previous *EPAS1* loss-of-function studies in animal models suggest a protective effect against chronic hypoxia, in which heterozygous knockout mice (*Epas1*^+/−^) exhibit lower [Hb] and reduced risk of pulmonary hypertension and right ventricular hypertrophy relative to wild-type mice following exposure to sustained hypoxia (*54*).

### *EPAS1* variant is associated with serum metabolites in Andeans

To investigate the relationship between circulating metabolites, Hct, and rs570553380, we completed analysis of serum metabolites via liquid chromatography–mass spectrometry (LC-MS) in a subset of the Quechua Andean cohort (*n* = 115). Of the 5957 metabolites examined from distinctive LC-MS peaks, 298 (21 previously identified, 277 unidentified) were associated with Hct after false discovery rate (FDR) correction (FDR < 0.05). When cross-compared with the FINRISK cohort, a Finnish population survey of chronic, noncommunicable diseases (*55*) (*n* = 1170), 12 of these 298 metabolites were associated with higher Hct in Andeans, a finding validated in FINRISK data (table S6).

**Fig. 4.**
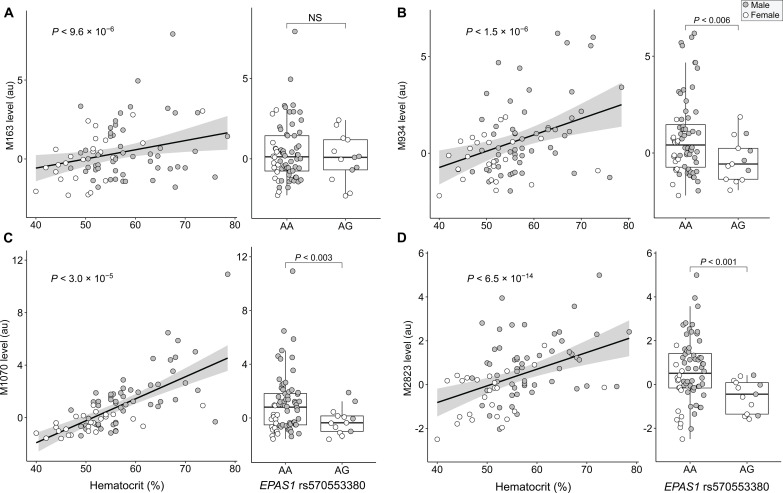
Metabolite associations with hematocrit and/or rs570553380 (A>G, p.[His194Arg]). (**A**) Hematocrit in the Andean cohort was associated with a previously identified metabolite (ID: M163) classified as endogenous cannabinoid arachidonoyl ethanolamide. Metabolites with putative IDs M934 (**B**), M1070 (**C**), and M2823 (**D**) were associated both with hematocrit and the *EPAS1* rs570553380 variant in the Andean cohort. These metabolites were found in a large-cohort population study (FINRISK) and are yet to be classified. Male and female data points are indicated with closed and open circles, respectively.

Among these 12 metabolites, endogenous cannabinoid arachidonoyl ethanolamide (M163) was the only previously identified metabolite associated with higher Hct (Fig. 4A). Of the 12 metabolites identified both in Andeans and FINRISK, only three (M934, M1070, and M2832) were further associated with rs570553380 in Andeans (Fig. 4, B to D). All three unidentified metabolites were negatively correlated with increased copies of the putatively adaptive G allele and positively correlated with Hct. Mendelian randomization indicated that Hct is most likely causal to these three metabolites, implicating their role in biological processes downstream of red blood cell production. The only previously identified metabolite associated with Hct in Andeans and FINRISK (M163) was not associated with rs570553380 in the Andean cohort (Fig. 4A).

## DISCUSSION

Associations between adaptive *EPAS1* variants and [Hb] in Tibetan groups at high altitude have been well replicated (*3*, *5*). Although overlapping signals of selection have been observed across Tibetans and Andeans (*12*, *25*), convergent adaptation involving the same genes and phenotypes has not been shown. Here, we demonstrate evidence for shared genotypic-phenotypic signals between Tibetan and Andean highlanders. Although the average [Hb] ([Hb] ∝ Hct) is notably lower in Tibetans compared to Andeans (*56*), Andeans with the positively selected *EPAS1* variant (rs570553380, A>G, p.[His194Arg]) demonstrate relatively lower Hct and protection from EE. Furthermore, this Andean-specific, protein-coding *EPAS1* variant attenuates activity of certain canonical HIF-2α targets under hypoxia, a molecular phenotype similarly associated with noncoding Tibetan-specific *EPAS1* variants (*11*, *53*, *54*).

In contrast to *EPAS1* variants that are found at high frequency and nearly fixed in many Tibetan groups (*3*), the Andean-specific variant presented here is observed at low frequency. Considering the notably young age (~9845 to ~13,027 years) and modest frequency of the Andean-specific variant, rs570553380(G) is likely under early-stage selection in highlander populations of the Andes, as identified and noted in Colla Andean highlanders (*25*). In contrast, the adaptive *EPAS1* haplotype identified in Tibetan groups was inherited from Denisovans more than 48,000 years ago (*26*, *57*, *58*). Furthermore, ancestors of populations in present-day Tibet resided at high altitudes much longer than populations in the Andes (*1*, *56*), allowing more time for natural selection at this locus to occur. Significant iSAFE, as well as EHH and iHS results provide strong evidence for selection in this Andean cohort, demonstrating that haplotypes containing the derived rs570553380 allele (G) are significantly longer than those with the ancestral allele (A). The modest frequency of this adaptive variant could explain the inability of prior selection scans to identify rs570553380 as an outlier at the genome-wide level (*10*, *12*, *18*–*20*, *24*, *27*, *28*), as only methods designed to detect recent positive selection in Andeans have identified rs570553380 (*25*).

The in vitro results suggest the protein-coding variant in *EPAS1* has broad effects beyond correlations with Hct, O2 saturation, and EE. Presence of the His194Arg variant is associated with decreased expression of *ADM* (adrenomedullin) and *VEGF* (vascular endothelial growth factor A), suggesting that rs570553380 may confer protection from pulmonary hypertension and right ventricular hypertrophy (*59*, *60*), as recently demonstrated in a CRISPR mouse model (*10*). The Andean *EPAS1* variant is also associated with decreased expression of *BHLE40* (basic helix-loop-helix family member E40), which prevents myogenic repair following ischemic injury (*61*), and *HILPDA* (hypoxia inducible lipid droplet associated), a mediator of intracellular fatty acid metabolism (*62*). Whether rs570553380 directly or indirectly affects these traits and biomarkers of functional relevance will require investigation from multiple angles. Such associations may or may not be immediately apparent; e.g., recent studies in Andean groups at high altitude show that variation in levels of erythropoietin, which stimulates red blood cell production, does not correlate with Hct (*31*). It is plausible that changes in gene expression involving this HIF-2α variant lead to pleiotropic effects and alterations at various steps of the O2 transport cascade with concomitant or compensatory effects that remain to be determined.

The association between rs570553380 and Hct in Andean highlanders parallels similar findings observed in Tibetans (*3*, *5*) and suggests that this variant may improve evolutionary fitness at high altitude. As noted in Tibetans, elevated [Hb] (or EE) is a hallmark of CMS (*63*, *64*), a debilitating disease more prevalent in males than females (*37*, *38*, *64*). Andean males with the adaptive *EPAS1* allele demonstrate significantly lower Hct, higher O2 saturations under conditions of hypoxia, and lower incidence of EE. The lack of significance in females could be explained by the notably reduced sample size compared to males (*n* = 139 males, 49 females) and inability to control for relevant covariates, such as estrogen levels, menstruation cycle, and menopause status (*33*, *39*). Considering that Hct and [Hb] levels are generally lower in females than males (*65*), it is also plausible that the effects of rs570553380 become more pronounced with increasing severity of EE or CMS. Molecularly, this could manifest as protection from chronic hyperactivation of the HIF pathway due to prolonged exposure to high-altitude hypoxia (*30*) and worsening pulmonary arterial hypertension (*64*). This disease severity–dependent relationship could also explain the lack of significance between [Hb] and rs570553380 in a recent study of Andean highlanders (*10*) with a younger average participant age (24 years, versus 41 years in this study). It is well documented that EE and CMS severity worsen notably with age (*64*), suggesting that the protective effects of rs570553380 may be more pronounced in older individuals.

Although the Andean-specific and Tibetan-specific alleles converge upon a similar phenotype, they likely manifest via distinct molecular and physiological changes. The adaptive *EPAS1* variants characterized in Tibetans are located within cis-regulatory elements that alter gene expression of *EPAS1* (*11*, *53*, *54*) and affect protein levels of HIF-2α, likely in a temporal- and cell-specific manner. In contrast, the missense *EPAS1* variant described here in Andeans is protein coding, altering the structure and function of HIF-2α itself (*10*). Despite these differences, both population-specific alleles appear to attenuate HIF-2 activity in some form. Recent work in murine models demonstrates that Tibetan-specific *EPAS1* variants are likely pleiotropic in nature (*11*). The observed associations between rs570553380, Hct, and unknown metabolites in Andeans suggest that this missense *EPAS1* variant may also alter additional phenotypes beyond Hct (or [Hb]) that may be more direct targets of selection. Although not correlated with rs570553380, endogenous cannabinoid arachidonoyl ethanolamide (anandamide) was positively associated with Hct in both Andeans and FINRISK. Anandamide has been observed to alter sleep (*66*), neuroprotection, and immunosuppression via transient receptor potential vanilloid type-1 receptor and nuclear peroxisome proliferator–activated receptors (*67*), suggesting the endocannabinoid system may play a role in mediating the effects of EE. However, the relationship between Hct (or [Hb]), downstream metabolites, EE, and red blood cell turnover in Tibetans has not been studied (*68*). Further cross-population studies between Hct and metabolites could help elucidate the similarities and differences in the production and destruction of erythrocytes in Andeans and Tibetans.

While we provide cross-population evidence for genotype-phenotype adaptation in high-altitude humans involving different variants within the same gene, natural selection at various *EPAS1* sites in other highland species has been observed (*31*, *32*). The presence of the same *EPAS1* amino acid substitution (H>R) in coelacanths poses questions of potential convergent evolution at this residue. Substantial adaptations in lung function and morphology since the Cretaceous period (*69*) between the extant coelacanth, *Latimeria,* and its extinct predecessor, *Axelrodichthys*, further suggest potential impacts of selective forces, in which this H>R substitution may have arisen independently and been advantageous in the coelacanth genetic background.

Although these findings provide a step forward in the understanding of hypoxia adaptations, there are various limitations of this work. The use of an in vitro cell culture model, with set tensions and time courses, fails to capture the dynamic chemical gradients that exist within a vascularized organ and the nuances of chronic hypoxia in highlanders. Furthermore, the use of HEK293T cells to model the transcriptional effects of rs570553380 on HIF-2 activity is not fully representative of the diverse spectrum of cell types within the human body. Despite these drawbacks, this study adds to our understanding of human evolution and has important implications for health and disease. Integrative genomic and physiological assessments with gene editing and multiomics analyses will continue to provide crucial insights into functional adaptive variation at high altitude (*70*), and natural selection within our species, with biological and clinical implications for hypoxia-related diseases and personalized medicine (*68*).

## MATERIALS AND METHODS

### Participant recruitment and criteria

The study was approved by the University of California, San Diego Human Research Protection Program (UC San Diego Project, nos. 140235 and 171772) and the Institutional Ethics Committee for Humans of Universidad Peruana Cayetano Heredia (CIEH-UPCH Project, no. 59285). All participants received and signed a detailed consent form in Spanish that explained the study. Males and nonpregnant females ages 19 to 65 of Quechua Andean ancestry living in Cerro de Pasco, Peru (4340 m), were recruited (*n* = 224), including individuals with and without CMS and EE [Hct ≥ 63% in males ([Hb] ≥ 21 g/dl) and ≥ 57% in females ([Hb] ≥ 19 g/dl)]. Ancestry was self-reported, and individuals self-identified as Quechua with both maternal and paternal grandparents resident at high altitude. Anyone with a history of cardiovascular/pulmonary disease besides CMS or EE was excluded from the study. Participants were also excluded if they had traveled to lower elevation (<3000 m) for more than 7 days in the 6 months before the study or had undergone phlebotomies and/or blood transfusions within 6 months before study.

### Whole-genome sequencing

We performed high-coverage whole-genome sequencing (WGS) analysis of DNA from 40 unrelated Quechua Andean men and women selected at random from the 224 recruited Andeans in this study on the Illumina HiSeqX sequencing platform (average and minimum call rate, 92.4 and 91.7%, respectively). An average of 2.86 Gb of sequencing data was aligned for each genome. We obtained an average of 30× coverage (76.0% of the genome) with 99.5% exonic coverage. Approximately 3.76 million of the total 12.51 million single-nucleotide variants (SNVs) were identified across each genome with 14.1% previously unidentified SNVs. For each sample, we identified an average of 188,852 SNVs across the whole exome, of which 0.29% were novel. As determined by Annotate Variation (ANNOVAR), there were 41,236 synonymous and 48,057 nonsynonymous SNVs (10,600 missense, 82 stopgain, and 10 stoploss on average).

### Principal components analysis and admixture

We used the program ADMIXTURE to determine the distribution of genetic ancestry among Andean participants from Cerro de Pasco (*71*). A single population was determined through ADMIXTURE analysis, determined by fivefold cross validation, for the 40 Andean genomes. We combined these data with the 1000 Genomes Yoruba in Ibadan, Nigeria (YRI), Japanese in Tokyo (JPT), Iberian population in Spain (IBS), Colombian (CLM), Mexican (MXL), and Peruvian (PEL) genomes and ran an unsupervised analysis to estimate the best fit among populations and the distribution of genetic ancestry in Andeans (*72*). According to the best fit (*K* = 4) mode, the Andean cohort’s ancestry reflects a small fraction resembling the component identified in an Iberian population from Spain (IBS) and a large non-IBS, likely Native American, component (fig. S1). This is further illustrated by a projection of the Andean samples onto a PC space created using data from several populations in the 1000 Genomes dataset: Northern Europeans from Utah (CEU), Iberian (IBS), PEL, CLM, MXL, CHB, JPT, and YRI (fig. S2). We also ran Flare (*73*) to determine local ancestry at the *EPAS1* gene region (chr2:46,524,546-46,613,836) (GRCh37/hg19 by NCBI Gene) plus 100 kb up- and down-stream of the *EPAS1* gene using the Thousand Genome Project phase 3 as the reference population. No significant admixture was noted at this locus.

### Selection analyses

We used the iSAFE to further examine a 1-Mb region surrounding the *EPAS1* SNV rs570553380 in the Andean whole genomes using default parameters (*34*). Results were visualized using Locuszoom (*74*). The Andean whole genomes were polarized against the ENSEMBL *Homo sapiens* GRCh37/hg19 ancestral allele file. EHH and iHS analyses were conducted on rs570553380, rs7559484, and rs7556828 in the Andean whole genomes via *rehh* (*75*) using default parameters.

### Dating of variants

The ages of rs570553380, rs7559484, and rs7556828 were determined using the joint clock configuration on Genealogical Estimation of Variant Age (GEVA), which incorporates 1000 Genomes phase 3 into their software (*76*). The age of rs570553380 was additionally estimated within the 40 Andean whole genomes by setting the EHH threshold (probability of homozygote = 0.05) and calculating the distance to either side from rs570553380 (*77*). All analyses assumed a generation length of 25 years.

### Genotyping

Venous blood samples (~10 ml) were collected in EDTA tubes (BD Vacutainer, BD, Franklin Lakes, NJ, USA). Buffy coat was isolated from whole blood via centrifugation and processed for storage in a cell lysis solution (Qiagen, Germantown, MD, USA) in Cerro de Pasco, Peru, and all samples were shipped to the University of California, San Diego. Genomic DNA was obtained from cell lysis and purified using a Gentra Puregene Blood Kit (Qiagen, Germantown, MD, USA). Genomic DNA samples were stored at 4°C for less than 1 year before genotyping. DNA samples were diluted to working stock concentrations of 5 ng/ul. DNA quality was verified with NanoDrop 2000 (Thermo Fisher Scientific, Waltham, MA USA, 260/280 absorbance ratios of 1.8 to 2.0 and 260/230 absorbance ratios of 2.0 to 2.2) and gel electrophoresis to ensure samples were not degraded. After whole-genome sequence data were analyzed, targeted SNV genotyping at the rs570553380 locus was completed using Applied Biosystems TaqMan SNV Genotyping Assays (Thermo Fisher Scientific, Waltham, MA, USA). Quantitative real-time polymerase chain reactions (PCRs) were conducted on a QuantStudio 3 (Applied Biosystems, Foster City, CA, USA) with a 0.1-ml of thermal block.

Genotyping calls were determined with the Applied Biosystems Genotyping Analysis Module. The presence of each allele was determined by the multicomponent amplification patterns of fluorescent VIC and FAM dyes in relation to a ROX reference dye. The real-time experimental analysis method was used with heterozygote-permitted autocalling. Each automatic call was verified visually by the experimenter.

### Phenotype analyses

Phenotypes of the recruited participants were obtained in Cerro de Pasco, Peru, in August 2015, December 2015, December 2016, December 2017, and March 2019. Methods and equipment for phenotype collection were consistent across trips. Height, weight, resting heart rate, and resting O2 saturation were collected during the initial screening session as participants were seated with their right arm elevated to heart level. O2 saturation (SpO2) under conditions of inspired O2 at 10% ($F_{IO_{2}}$ = 0.10) was obtained in a subset of individuals as previously described (*40*). All O2 saturation and heart rate measurements were collected via pulse oximeter (Nellcor model N-560, Medtronic, MN, USA). Hematocrit (Hct), the proportion of red blood cells in whole blood, was measured from venous blood using capillary tubes. Individuals with a Hct ≥ 63% in men ([Hb] ≥ 21 g/dl) and ≥ 57% in women ([Hb] ≥ 19 g/dl) were considered to have EE (*63*).

### Genotype-phenotype associations

A multivariate generalized linear model was used to assess associations between *EPAS1* genotypes and phenotypic measurements of unrelated individuals. We collected self-reported information regarding familial relationships and excluded all but one of any related individuals. Our WGS analyses in 40 individuals confirmed self-reported nonrelatedness and ancestry. The regression model accounts for the various plausible modes of inheritance of potential genetic variants and can be written as:

$$\mathrm{Phenotype}=\beta_{0}+\beta_{G}\mathrm{Genotype}+\beta_{\mathrm{Age}}\mathrm{Age}+\beta_{\mathrm{Study}}\mathrm{Study}$$

where *Phenotype* is a continuous variable stratified by sex, *Genotype* is a factor with n levels (*n* ∈ {2,3}) dependent on the zygosity of the variant, *Age* and *Study* are covariates whose fixed and random effects were controlled. Age and study batch effects of measurements collected in *m* studies were no longer considered by the model if their corresponding coefficients were not significantly different from zero. A robust SW estimator was used to account for heteroskedasticity of the phenotypic data (*78*).

### Constructs and molecular cloning

Guide RNA (gRNA) expression plasmids with the spacers GGTCTTGCACTGCACGGGCC (for *EPAS1* H194R editing) and CAGTCAGCCGGTGGTGCAGA (for deadEGFP editing) were generated via site-directed mutagenesis cloning using pFYF1230 (Addgene plasmid no. 47511) as a template and Phusion High-Fidelity DNA Polymerase (Thermo Fisher Scientific, no. F534L) according to the manufacturer’s instructions. All DNA vector amplification was carried out using NEB 10-β competent cells (NEB, no. C3019H). All plasmids were purified using the ZymoPURE II Plasmid Midiprep Kit (Zymo Research, no. 11-550B).

### HEK293T cell culture and transfections for base editing experiments

HEK293T cells (ATCC CRL-3216) were cultured and maintained in high-glucose Dulbecco’s modified Eagle’s medium (DMEM) supplemented with GlutaMAX (Thermo Fisher Scientific, no. 10566024), 10% (v/v) fetal bovine serum (Life Technologies, no. 10437-028), and penicillin-streptomycin (P/S) (100 U/ml; Gibco, no. 15070063), at 37°C with 5% CO2. Transfection of plasmid DNA into HEK293T cells for the purpose of generating isogenic cell lines has been previously described (*79*). Briefly, cells were plated at a density of 50,000 cells per well in 250 μl of P/S-free medium per well in 48-well plates and transfected 16 hours after plating. For each transfection, 200 ng of gRNA plasmid (described above) and 800 ng of base editor plasmid (pABEmax-NG-P2A-EGFP, Addgene, no. 140005) were combined with 1.5 μl of Lipofectamine 2000 (Life Technologies, no. 11668-019) and Opti-MEM (Life Technologies, no. 31985070) according to the manufacturer’s instructions. For the generation of the *EPAS1* cell lines in this study, cells were sorted to enrich for high base-editing activity using a mCherry-P2A-deadEGFP plasmid. In these transfections, 300 ng of the mCherry-P2A-deadEGFP plasmid and 100 ng of its corresponding gRNA expressing plasmid were included in the lipofectamine mixture. A similar technique has been previously described (*80*). Briefly, a successful A•T to G•C edit within a nonfluorescent EGFP results in GFP fluorescence, allowing for fluorescence-activated cell sorting (FACS) enrichment.

### Sequencing to assess base editing efficiencies

Cells were cultured for 3 days following transfection. Genomic DNA was extracted after rinsing with 150 μl of phosphate-buffered saline (PBS, Gibco Life, no. 10010-023) with 100 μl of freshly prepared cell lysis solution [10 mM tris-HCl (pH 7.5), 0.05% SDS, and proteinase K (25 μg/ml; NEB, no. P8107S)] for each well. The cell lysate was transferred to 0.2-ml PCR tubes and incubated at 37°C for 1 hour and then heat-inactivated at 80°C for 30 min. This cell lysate (0.5 μl; gDNA) was used as a PCR template with the forward primer ATCGTGAGGCTGCTGGACGAGT and the reverse primer TCTCCACTCAGGAAGCTCCGGC. The PCR amplicon was sequenced with Sanger sequencing using the forward primer. After confirmation of >10% A•T to G•C editing efficiency at H194 of the *EPAS1* genomic locus using EditR (*81*), the transfection step was repeated. However, instead of harvesting the cells after 3 days, they were sorted using FACS and allowed to clonally expand as described below.

### Utilization of FACS to isolate base-edited single-cell *EPAS1* H194R clones

Three days after transfection, the old medium was aspirated off and the cells were rinsed with 150 μl of PBS. The cells were then detached from the plate with 300 μl of Accumax (Innovative-Cell Technology, no. AM-105) and resuspended with the addition of 500 μl of PBS. The cell suspension was transferred to a 15-ml conical tube, which was centrifuged for 5 min at 200 rcf at room temperature. After aspirating off the supernatant, the cells were resuspended in FACS buffer [1% fetal bovine serum (FBS), 50 μM EDTA (pH 8.0), and propidium iodide (PI) (2 μg/ml; Sigma-Aldrich, no. P3566)] and filtered through a cell strainer. Using a FACSAria II system, nonviable cells and doublets were eliminated via gating parameters and under sterile conditions, single cells fluorescent in both mCherry and EGFP were sorted into individual wells of 96-well plates containing 100 μl of DMEM supplemented with 50% FBS and 1% Pen/Strep. The 96-well plate was immediately placed into a tissue culture incubator after sorting. Subculturing and genotyping of single cells after clonal expansion was conducted as previously described (*79*). Genotyping of 26 clones confirmed the presence of three clones containing a heterozygous genotype and one clone containing a homozygous genotype (fig. S7). Heterozygotes were defined as containing at least one mutant and one wild-type allele. These, along with two unedited clones, were cryopreserved and subjected to further experiments.

### WES analysis of edited cell lines

Whole-exome sequencing (WES) data for a subset of the isogenic cell lines ran in duplicate were generated on the Illumina NovaSeq 6000 (DNA Link Precision Genomics, Seoul Korea). Raw WES data were aligned to the reference human genome (hg19) using the Burrows-Wheeler aligner (*82*), and variants of the aligned genome were called using Freebayes (*83*). Variants with call quality scores ≤50 and/or inconsistent calls across runs were excluded. Variant call files were then annotated using Qiagen Clinical Insight (Qiagen, Germantown, MD, USA) (table S4). To assess relative enrichment of off-target mutations across cell lines (fig. S8A), the mutation rates of all observed SNVs were compared to those of the nontransfected control (fig. S8B).

### In vitro hypoxia experiments

For all hypoxia experiments, low passage, isogenic clones wild-type (WT), heterozygous (HET), and homozygous (HOM) for the *EPAS1* variant (rs570553380, A>G, p.[His194Arg]) were used within several passages of defrosting from cryopreservation. Data from the single HOM clone are shown but not included in statistical analyses. Both wild-type HEK293T cells that had [wild-type transfected (WTT)] and had not [wild-type nontransfected (WTNT)] undergone the transfection and clonal expansion process were used as controls. Cells were grown within a 37°C humidified incubator (Heracell VIOS 160i Tri-Gas CO2, Thermo Fisher Scientific) under hypoxia (1% O2) and normoxia (21% O2) for 0, 12, and 24 hours. The use of a cell culture model system with O2 exposure ranging from room air to as low as 0.5% provides a powerful tool to study cellular mechanisms in hypoxia. The selection of 1% O2 was based off prior studies in which O2 tension induced HIF-2 stabilization in HEK293T cells (*49*–*52*). O2 and CO2 levels were calibrated before experimentation against external sensors. Pericellular dissolved O2 and pH levels were measured every 5 min using the sbi ID Developer’s Kit and Data Hub (Scientific Bioprocessing, Pittsburgh, PA, USA). For each time point (normoxia, 12 hours, 24 hours), total RNA and protein were immediately extracted for downstream applications.

### RNA extraction and quantitative real-time PCR

Following a 2-ml PBS wash to remove dead cells and waste, total RNA was immediately extracted for all time points (normoxia, 12 hours, 24 hours) using the RNeasy Mini kit with DNase digestion according to manufacturer’s instructions (Qiagen, Germantown, MD, USA). After extraction, RNA concentration and quality were measured via NanoDrop 2000 (Thermo Fisher Scientific, Waltham, MA USA). Total RNA (1 μg) was then converted into cDNA using the High-Capacity cDNA Reverse Transcriptase kit (Thermo Fisher Scientific, Waltham, MA, USA) according to the manufacturer’s instructions.

Quantitative real-time PCR was carried out in duplicate for all biological samples on the QuantStudio 3 (Applied Biosystems, Foster City, CA, USA) with a 0.1-ml thermal block using the TaqMan Fast Advanced Master Mix (Thermo Fisher Scientific, Waltham, MA, USA). TaqMan assays (Thermo Fisher Scientific) for canonical HIF-2α target genes (table S5) were normalized to β-actin (*ACTNB*) as a housekeeping gene in all edited cell lines. The ΔΔCt method was conducted to calculate differential gene expression of each isogenic cell line at all three time points normalized by their normoxia counterparts, where ΔCt = Ct target − Ctβ-actin. Relative expression was then quantified by ΔΔCt = ΔCt hypoxia − ΔCt normoxia and then compared between nontransfected and transfected wild-type cell lines as well as between genotypes using a two-way analysis of variance (ANOVA) test. The genes used for quantitative real-time PCR (table S5) were assayed in prior studies assessing HIF-2 activity (*84*–*87*).

### Protein extraction and Western blot analysis

HEK293 cells cultured in T25 flasks were exposed to normoxia (21% O2) or 24-hour hypoxia (1% O2). At time of collection, flasks were rapidly taken out from the incubator, cell media was discarded, and cells were washed with 1.6 ml of chilled PBS. Cells were retrieved with a cell scraper in 600 μl of radioimmunoprecipitation assay buffer (no. 9806S, Cell Signaling Technology, MA, USA) with 0.1% of Protease Inhibitor Cocktail (no. P8340, Sigma-Aldrich, USA) and collected in Eppendorf tubes. For lysis, the cells were incubated on ice for 15 min with successive vortex agitation cycles and then stored at - 80°C. Protein content was determined using DC Protein Assay (BioRad, CA, USA). Proteins were separated in 10% Bis Tris-Plus premade gels (Mini Gel Tank using Bolt buffers, Thermo Fisher Scientific, Carlsbad, CA), and transferred to polyvinyldene difluoride membranes (iBlot, Invitrogen, Carlsbad, CA). Membranes were blocked for 1 hour with 5% bovine serum albumin (no. A-7030, Sigma-Aldrich, USA), diluted in PBS with 0.1% Tween 20 at room temperature, and then incubated with primary antibodies against HIF-2α (Novus, no. NB-100-122, 1/250) and β-actin (Cell Signaling Technology, no. 4970S, 1/1000) overnight at 4°C, washed, and incubated with fluorescent secondary antibodies for imaging detection (Odyssey Infrared Imager, LiCOR). Protein band quantification was performed with ImageJ software. Data are presented as the ratio of HIF-2α to β-actin band intensities.

### Metabolomics and association validation

To understand the upstream and downstream metabolomic effects on high-altitude adaptation, we analyzed 116 plasma samples from the subset of the full Andean cohort, which consisted of 59 men and 57 women. LC-MS was used to identify unique peaks suggesting the existence/abundance of previously identified and unidentified metabolites (*88*). Metabolite signals were standardized by dividing the peak-median intensity difference by the median absolute deviation. To test for metabolite-phenotype associations, we used a generalized linear model defined as

$$M=\beta_{0}+\beta_{P}\mathrm{Phe}+\beta_{\mathrm{Sex}}\mathrm{Sex}+\beta_{\mathrm{Age}}\mathrm{Age}+\beta_{\mathrm{BMI}}\mathrm{BMI}$$

where *M* is the standardized signal of metabolite level, *Phe* refers to the measurements of hematocrit (%), and sex, age, and body mass index (BMI) were considered as covariates. We controlled for sex in these analyses for direct comparison to publicly available metabolomics data from FINRISK. FDR correction was conducted based off the number of distinct LC-MS peaks detected. Furthermore, associations of the top metabolites revealed in the Andean cohort were validated using the FINRISK cohort (*n* = 1170) with the same model. Mendelian randomization was additionally performed (*89*) on the significant hits to infer statistical causality.

### Molecular graphics representations

The ribbon diagram of HIF-2α was generated on IBS software (*90*), while the structure of HIF-2α [Protein Data Bank (PDB) ID: 4ZPK] was annotated on UCSF Chimera (*91*).

### Structural analysis and prediction of rs570553380

Analysis of rs570553380 on the structure and stability of HIF-2α (PDB ID: 4ZP4) was conducted using PROVEAN (*45*), MutPred2 (*46*), and DynaMut2 (*47*).
