# Supplementary material for: Functional EPAS1/HIF2A missense variant is associated with hematocrit in Andean highlanders
Source: Sci Adv. 2024 Feb 9;10(6):eadj5661. doi: 10.1126/sciadv.adj5661 (PMC10857371; doi:10.1126/sciadv.adj5661)
Supplement: Supplementary file 1 — Figs. S1 to S9 Tables S1 to S3 and S5 Legends for tables S4 and S6 [file sciadv.adj5661_sm.pdf]

Science Advances
AAAS

Supplementary Materials for

Functional *EPAS1*/*HIF2A* missense variant is associated with hematocrit in Andean highlanders

Elijah S. Lawrence *et al.*

Corresponding author: Tatum S. Simonson, tsimonson@ucsd.edu

Sci. Adv. **10**, eadj5661 (2024)
DOI: 10.1126/sciadv.adj5661

The PDF file includes:

Figs. S1 to S9
Tables S1 to S3 and S5
Legends for tables S4 and S6

Other Supplementary Material for this manuscript includes the following:

Tables S4 and S6

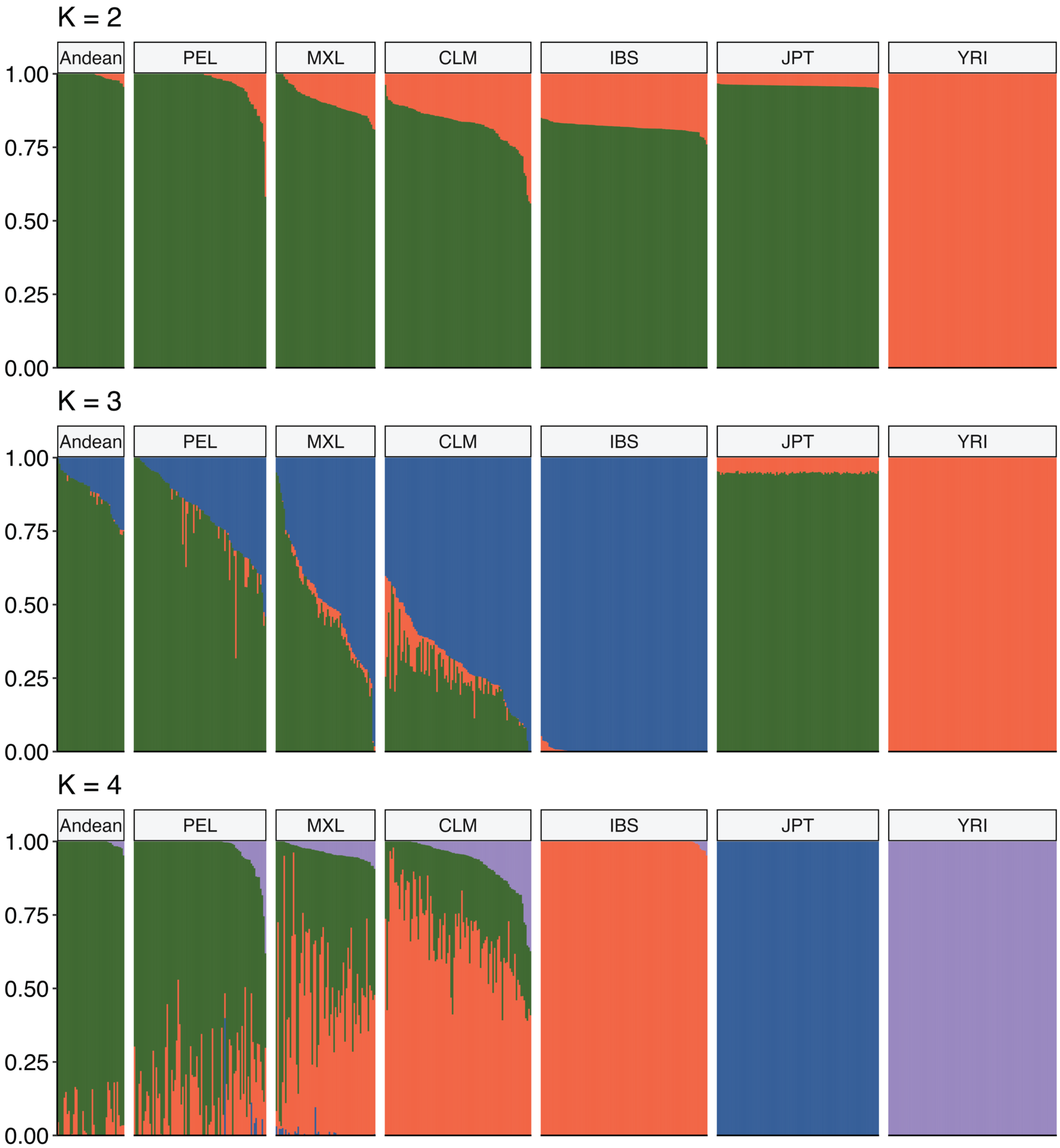

Fig. S1. K clustering plot of Andean cohort with 1000 Genomes sub-populations. We used the program ADMIXTURE to assess ancestry components in the Andean cohort from Cerro de Pasco, Peru, relative to Peruvians from Lima (PEL), Mexican (MXL), Columbian (CLM), Iberian (IBS), Japanese (JPT), and Yoruba (YRI) sub-populations from 1000 Genomes Phase III for a K range of 1 to 10. Each individual genome is represented as a vertical bar and grouped by population as shown (best fit by cross validation error at K = 4). A small proportion (~5.21%) of Andean ancestry is similar to the component represented in all IBS individuals and at increasingly higher proportions among individuals from PEL, MXL, and CLM populations.

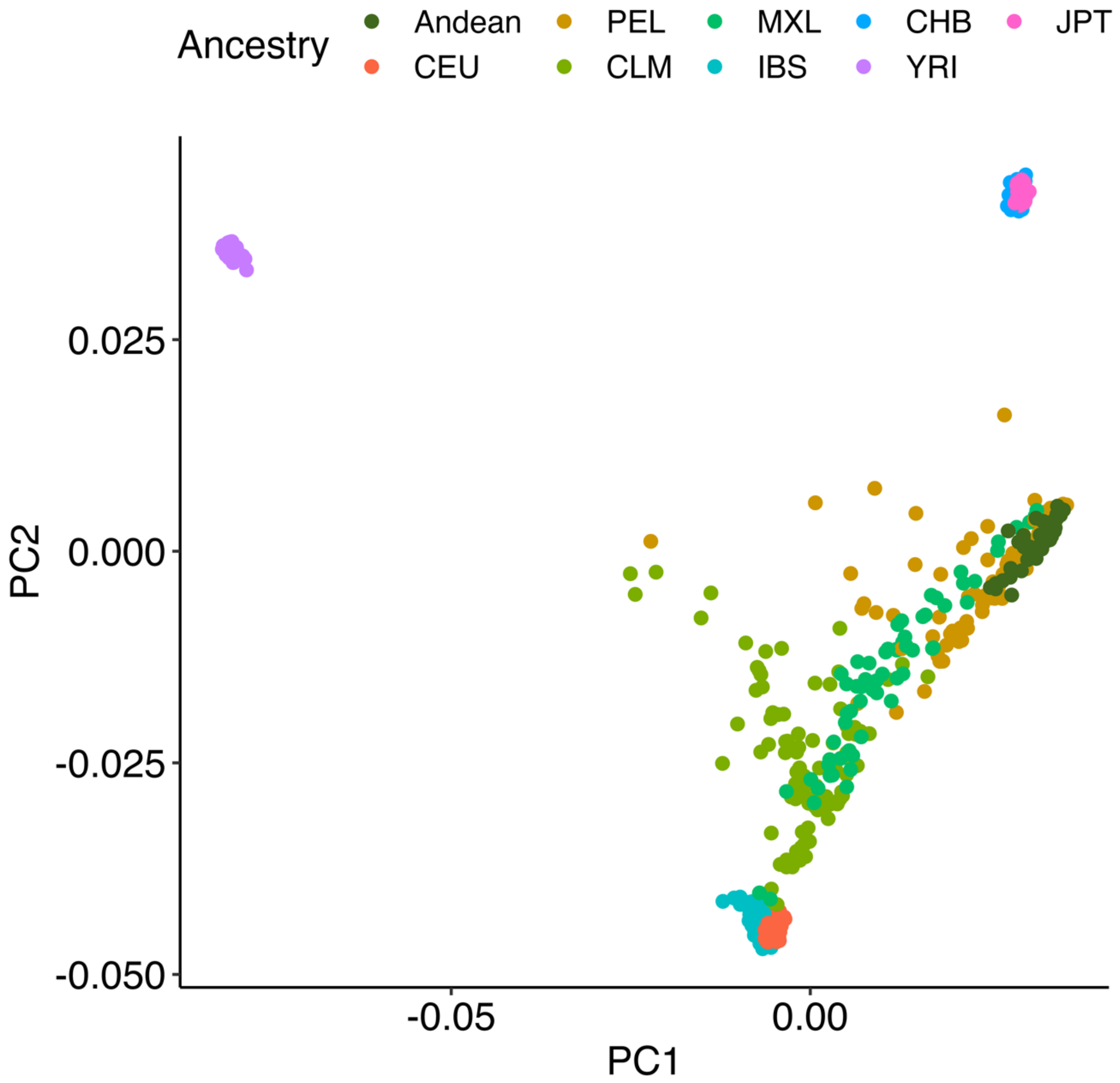

Fig. S2. PC1 and PC2 from Principal Components Analysis (PCA) of Andean cohort genomes and 1000 Genomes sub-populations. To assess genetic similarity between groups, we conducted Principal Components Analysis (PCA) of the Andean samples and Northern and Western European (CEU), Colombian (CLM), Mexican (MXL), Peruvian (PEL), Iberian (IBS), Han Chinese (CHB), Yoruba (YRI), and Japanese (JPT) sub-populations from 1000 Genomes Phase III. Data from Andean individuals cluster distantly from the CEU and IBS cluster along the axis shared with CLM, MXL, and PEL. PC1, PC2, and PC3 explain 49%, 26%, and 10% of the variance, respectively, while PCs 4 through 10 all explain <1%.

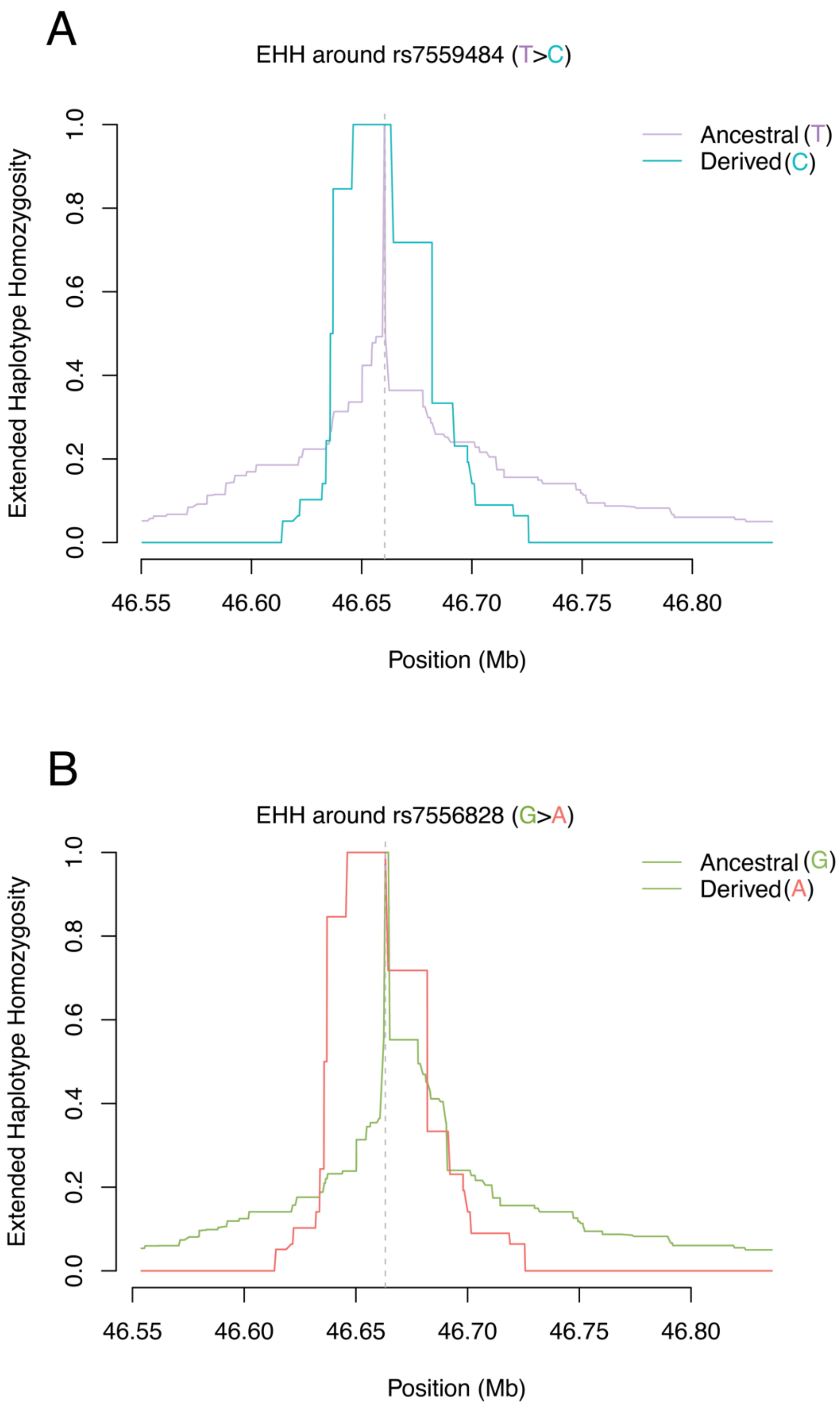

Fig. S3. Extended haplotype homozygosity (EHH) plots of rs7559484 and rs7556828 in the Quechuan Andean whole genomes. EHH and Integrated haplotype scores (iHS) for rs7559484 and rs7556828 were insignificant for both variants (iHS = -0.62, $p > 0.53$ for both).

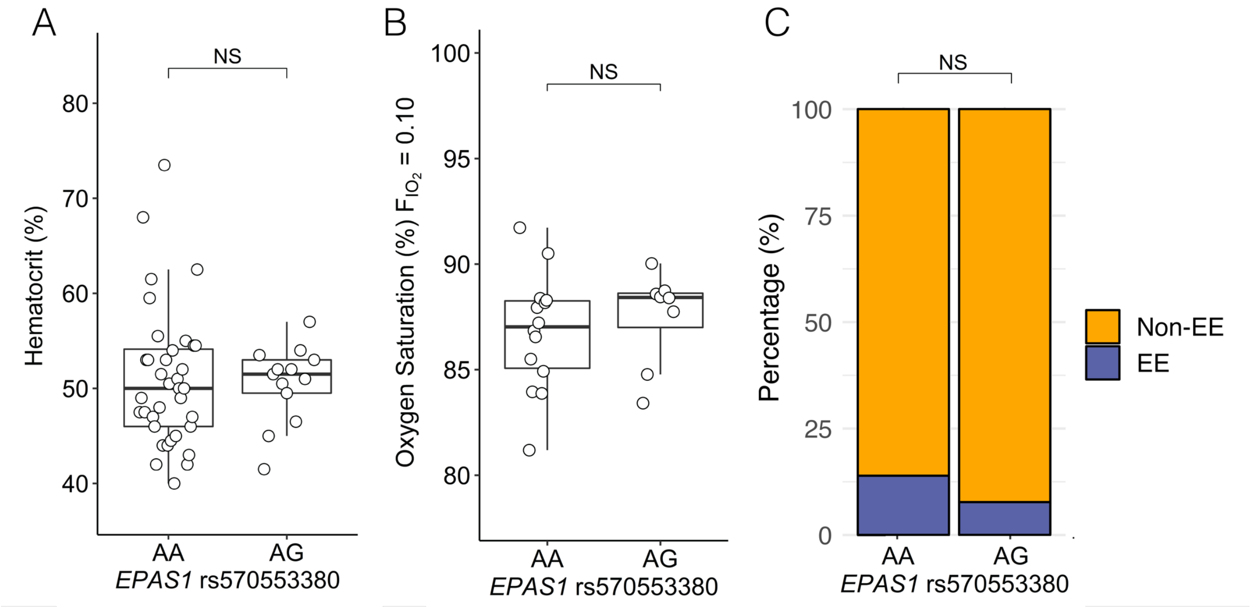

Fig. S4. Positively selected *EPAS1/HIF2A* variant is not associated with hematocrit, oxygen saturation, nor incidence of excessive erythrocytosis (EE) in Andean females. The *EPAS1* variant (rs570553380, A>G, p.[His194Arg]) was not significantly correlated with Hct (%) (n = 49) (A), oxygen saturation (SpO_2) at F_{IO_2}= 0.10 (n = 22, data from (*40*)) (B), nor incidence of EE (n = 49) (C) in Andean females.

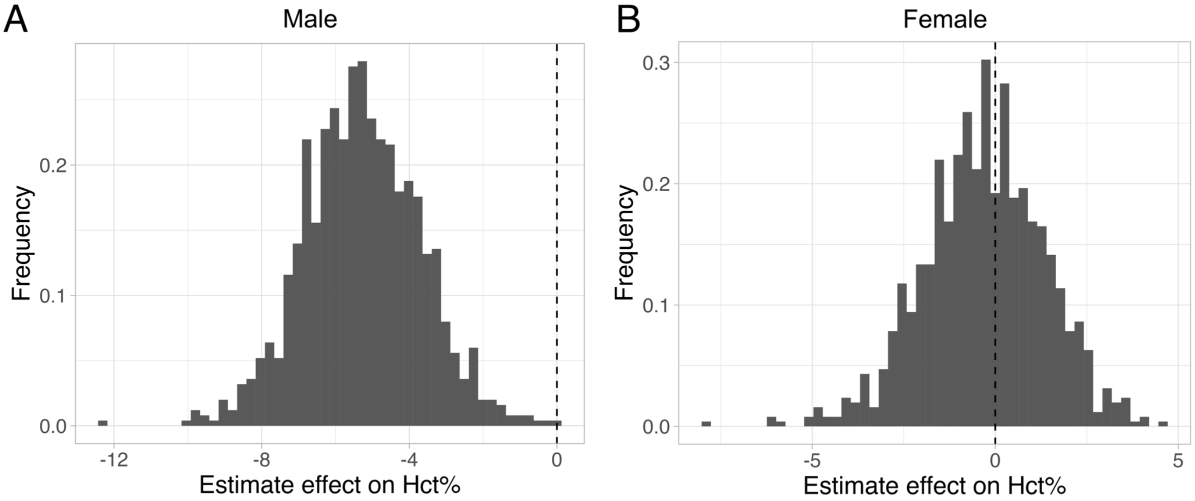

Fig. S5. Permutation test on rs570553380 association with percent hematocrit in Andean males and females. The *EPAS1* variant (rs570553380, A>G, p.[His194Arg]) was tested against percent hematocrit (Hct%) in a simulated dataset 1,000 times each in males (A) and females (B) based off the experimental population (n = 139 males, n = 49 females). The distribution of rs570553380's effect on Hct was determined to have a median and mean of -5.3% and -5.2% (95% CI: [-8.6, -2.0]) in males and -0.2% and -0.4% (95% CI: [-3.9, 2.5]) in females. In males, the simulated estimated effect is significantly deviated from zero. However, in females, the simulated estimated effect is not significantly deviated from zero.

		EXON5 186	EXON5 187	EXON5 188	EXON5 189	EXON5 190	EXON5 191	EXON6 192	EXON6 193	EXON6 194	EXON6 195	EXON6 196	EXON6 197	EXON6 198	EXON6 199	EXON6 200	EXON6 201	EXON6 202	EXON6 203
**Primate subset**																			
**Human**	**Homo sapiens**	**K**	**S**	**A**	**T**	**W**	**K**	**V**	**L**	**H**	**C**	**T**	**G**	**Q**	**V**	**K**	**V**	**Y**	**N**
Chimp	Pan troglodytes	--	--	--	--	--	--	--	--	--	--	--	--	--	--	--	--	--	--
Orangutan	Pongo pygmaeus abelii	--	--	--	--	--	--	--	--	--	--	--	--	--	--	--	--	--	--
Gibbon	Nomascus leucogenys	--	--	--	--	--	--	--	--	--	--	--	--	--	--	--	--	--	--
Rhesus	Macaca mulatta	--	--	--	--	--	--	--	--	--	--	--	--	--	--	--	--	--	--
Crab-eating macaque	Macaca fascicularis	--	--	--	--	--	--	--	--	--	--	--	--	--	--	--	--	--	--
Baboon	Papio hamadryas	--	--	--	--	--	--	--	--	--	--	--	--	--	--	--	--	--	--
Green monkey	Chlorocebus sabaeus	--	--	--	--	--	--	--	--	--	--	--	--	--	--	--	--	--	--
Marmoset	Callithrix jacchus	--	--	--	--	--	--	--	--	--	--	--	--	--	--	--	--	--	--
Squirrel monkey	Saimiri boliviensis	--	--	--	--	--	--	--	--	--	--	--	--	--	--	--	--	--	--
Bushbaby	Otolemur garnettii	--	--	--	--	--	--	--	--	--	--	--	--	--	--	--	--	--	D
**Euarchontoglires subset**																			
Chinese tree shrew	Tupaia chinensis	--	--	--	--	--	--	--	--	--	--	--	--	--	--	--	--	--	--
Squirrel	Spermophilus tridecemlineatus	--	--	--	--	--	--	--	--	--	--	--	--	--	--	--	--	--	--
Lesser Egyptian jerboa	Jaculus jaculus	--	--	--	--	--	--	--	--	--	--	--	--	--	--	--	--	C	--
Prairie vole	Microtus ochrogaster	--	--	--	--	--	--	--	--	--	--	--	--	--	--	R	--	--	--
Chinese hamster	Cricetulus griseus	--	--	--	--	--	--	--	--	--	--	--	--	--	--	R	--	--	--
Mouse	Mus musculus	--	--	--	--	--	--	--	--	--	--	--	--	--	--	R	--	--	--
Rat	Rattus norvegicus	--	--	--	--	--	--	--	--	--	--	--	--	--	--	R	--	--	--
Naked mole-rat	Heterocephalus glaber	--	--	--	--	--	--	--	--	--	--	--	--	--	--	--	--	--	--
Guinea pig	Cavia porcellus	--	--	--	--	--	--	--	--	--	--	--	--	--	--	--	--	--	--
Chinchilla	Chinchilla lanigera	--	--	--	--	--	--	--	--	--	--	--	--	--	--	--	--	--	--
Brush-tailed rat	Octodon degus	--	--	--	--	--	--	--	--	--	--	--	--	--	--	--	--	--	--
Rabbit	Oryctolagus cuniculus	--	--	--	--	--	--	--	--	--	--	--	--	--	--	--	--	--	--
Pika	Ochotona princeps	--	--	--	--	--	--	--	--	--	--	--	--	--	--	--	--	--	S
**Laurasiatheria subset**																			
Pig	Sus scrofa	--	--	--	--	--	--	--	--	--	--	--	--	--	--	--	--	--	--
Alpaca	Vicugna pacos	--	--	--	--	--	--	--	--	--	--	--	--	--	--	--	--	--	--
Bactrian camel	Camelus ferus	--	--	--	--	--	--	--	--	--	--	--	--	--	--	--	--	--	--
Dolphin	Tursiops truncatus	--	--	--	--	--	--	--	--	--	--	--	--	--	--	--	--	--	--
Killer whale	Orcinus orca	--	--	--	--	--	--	--	--	--	--	--	--	--	--	--	--	--	--
Cow	Bos taurus	--	--	--	--	--	--	--	--	--	--	--	--	--	--	--	--	--	--
Sheep	Ovis aries	--	--	--	--	--	--	--	--	--	--	--	--	--	--	--	--	--	S
Domestic goat	Capra hircus	--	--	--	--	--	--	--	--	--	--	--	--	--	--	--	--	--	S
Horse	Equus caballus	--	--	--	--	--	--	--	--	--	--	--	--	--	--	--	--	--	--
White rhinoceros	Ceratotherium simum	--	--	--	--	--	--	--	--	--	--	--	--	--	--	--	--	--	--
Cat	Felis catus	--	--	--	--	--	--	--	--	--	--	--	--	--	--	--	--	--	--
Dog	Canis lupus familiaris	--	--	--	--	--	--	--	--	--	--	--	--	--	--	--	--	--	--
Ferret	Mustela putorius furo	--	--	--	--	--	--	--	--	--	--	--	--	--	--	--	--	--	S
Panda	Ailuropoda melanoleuca	--	--	--	--	--	--	--	--	--	--	--	--	--	--	--	--	--	--
Pacific walrus	Odobenus rosmarus divergens	--	--	--	--	--	--	--	--	--	--	--	--	--	--	--	--	--	--
Weddell seal	Leptonychotes weddellii	--	--	--	--	--	--	--	--	--	--	--	--	--	--	--	--	--	S
Black flying-fox	Pteropus alecto	--	--	--	--	--	--	--	--	--	--	--	--	--	--	--	--	--	--
Megabat	Pteropus vampyrus	--	--	--	--	--	--	--	--	--	--	--	--	--	--	--	--	--	--
David's myotis bat	Myotis davidii	--	--	--	--	--	--	--	--	--	--	--	--	--	--	--	--	--	--
Microbat	Myotis lucifugus	--	--	--	--	--	--	--	--	--	--	--	--	--	--	--	--	--	--
Big brown bat	Eptesicus fuscus	--	--	--	--	--	--	--	--	--	--	--	--	--	--	--	--	--	--
Hedgehog	Erinaceus europaeus	--	--	--	--	--	--	--	--	--	--	--	--	--	--	--	--	--	--
Shrew	Sorex araneus	--	--	--	--	--	--	--	--	--	--	--	--	--	--	--	--	--	S
Star-nosed mole	Condylura cristata	--	--	--	--	--	--	--	--	--	--	--	--	--	--	--	--	--	S
**Afrotheria subset**																			
Elephant	Loxodonta africana	--	--	--	--	--	--	--	--	--	--	--	--	--	M	--	--	--	--
Cape elephant shrew	Elephantulus edwardii	--	--	--	--	--	--	--	--	--	--	--	--	--	M	--	--	--	--
Manatee	Trichechus manatus latirostris	--	--	--	--	--	--	--	--	--	--	--	--	--	M	--	--	--	--
Cape golden mole	Chrysochloris asiatica	--	--	--	--	--	--	--	--	--	--	--	--	--	M	--	--	--	--
Tenrec	Echinops telfairi	--	--	--	--	--	--	--	--	--	--	--	--	--	M	--	--	--	--
Aardvark	Orycteropus afer afer	--	--	--	--	--	--	--	--	--	--	--	--	--	M	--	--	--	--
Mammal subset		--	--	--	--	--	--	--	--	--	--	--	--	--	--	--	--	--	--
Armadillo	Dasypus novemcinctus	--	--	--	--	--	--	--	--	--	--	--	--	--	--	--	--	--	--
Opossum	Monodelphis domestica	--	--	--	--	--	--	--	--	--	--	--	--	--	--	--	--	--	--
Tasmanian devil	Sarcophilus harrisii	--	--	--	--	--	--	--	--	--	--	--	--	--	--	--	--	--	--
Wallaby	Macropus eugenii	--	--	--	--	--	--	--	--	--	--	--	--	--	--	--	--	--	--
**Aves subset**																			
Saker falcon	Falco cherrug	--	--	--	--	--	--	--	--	--	--	--	--	--	--	--	--	--	--
Peregrine falcon	Falco peregrinus	--	--	--	--	--	--	--	--	--	--	--	--	--	--	--	--	--	--
Collared flycatcher	Ficedula albicollis	--	--	--	--	--	--	--	--	--	--	--	--	--	--	--	--	--	--
White-throated sparrow	Zonotrichia albicollis	--	--	--	--	--	--	--	--	--	--	--	--	--	--	--	--	--	--
Medium ground finch	Geospiza fortis	--	--	--	--	--	--	--	--	--	--	--	--	--	--	--	--	--	--
Zebra finch	Taeniopygia guttata	--	--	--	--	--	--	--	--	--	--	--	--	--	--	--	--	--	--
Tibetan ground jay	Pseudopodoces humilis	--	--	--	--	--	--	--	--	--	--	--	--	--	--	--	--	--	--
Budgerigar	Melopsittacus undulatus	--	--	--	--	--	--	--	--	--	--	--	--	--	--	--	--	--	--
Parrot	Amazona vittata	--	--	--	--	--	--	--	--	--	--	--	--	--	--	--	--	--	--
Scarlet macaw	Ara macao	--	--	--	--	--	--	--	--	--	--	--	--	--	--	--	--	--	--
Rock pigeon	Columba livia	--	--	--	--	--	--	--	--	--	--	--	--	--	--	--	--	--	--
Mallard duck	Anas platyrhynchos	--	--	--	--	--	--	--	--	--	--	--	--	--	--	--	--	--	--
Chicken	Gallus gallus	--	--	--	--	--	--	--	--	--	--	--	--	--	--	--	--	--	--
Turkey	Meleagris gallopavo	--	--	--	--	--	--	--	--	--	--	--	--	--	--	--	--	--	--
**Sarcopterygii subset**																			
American alligator	Alligator mississippiensis	--	--	--	--	--	--	--	--	--	--	--	--	--	--	--	--	--	--
Green seaturtle	Chelonia mydas	--	--	--	--	--	--	--	--	--	--	--	--	--	--	--	--	--	--
Painted turtle	Chrysemys picta bellii	--	--	--	--	--	--	--	--	--	--	--	--	--	--	--	--	--	--
Chinese softshell turtle	Pelodiscus sinensis	--	--	--	--	--	--	--	--	--	--	--	--	--	--	--	--	--	--
Spiny softshell turtle	Apalone spinifera	--	--	--	--	--	--	--	--	--	--	--	--	H	--	R	--	--	D
Lizard	Anolis carolinensis	--	--	--	--	--	--	--	--	--	--	--	--	--	I	--	--	--	--
X. tropicalis	Xenopus tropicalis	--	--	--	--	--	--	--	--	--	--	--	--	H	--	--	A	--	--
Coelacanth	Latimeria chalumnae	--	--	--	--	--	--	--	--	R	--	--	--	H	I	--	--	--	--
**Fish subset**																			
Tetraodon	Tetraodon nigroviridis	--	--	--	S	--	--	--	--	--	--	--	--	--	L	--	M	--	--
Fugu	Takifugu rubripes	--	--	--	G	--	--	--	--	--	--	--	--	--	L	--	M	--	--
Yellowbelly pufferfish	Takifugu flavidus	--	--	--	G	--	--	--	--	--	--	--	--	--	L	--	M	--	--
Nile tilapia	Oreochromis niloticus	--	--	--	S	--	--	--	--	--	--	--	--	--	L	R	M	--	--
Princess of Burundi	Neolamprologus brichardi	--	--	--	S	--	--	--	--	--	--	--	--	--	L	R	M	--	--
Burton's mouthbreeder	Haplochromis burtoni	--	--	--	--	--	--	--	--	--	--	--	--	H	--	R	--	--	D
Zebra mbuna	Maylandia zebra	--	--	--	S	--	--	--	--	--	--	--	--	--	L	R	M	--	--
Pundamilia nyererei	Pundamilia nyererei	--	--	--	S	--	--	--	--	--	--	--	--	--	L	R	M	--	--
Medaka	Oryzias latipes	--	--	--	G	--	--	--	--	--	--	--	--	H	L	--	M	--	D
Southern platyfish	Xiphophorus maculatus	--	--	--	S	--	--	--	--	--	--	--	--	--	L	--	M	--	D
Stickleback	Gasterosteus aculeatus	--	--	--	S	--	--	--	--	--	--	--	--	H	L	--	M	--	S
Zebrafish	Danio rerio	--	--	--	S	--	--	--	--	--	--	--	--	H	L	Q	--	C	S
Mexican tetra (cavefish)	Astyanax mexicanus	--	--	--	S	--	--	--	--	--	--	--	--	H	L	--	--	--	--
Spotted gar	Lepisosteus oculatus	--	--	--	S	--	--	--	--	--	--	--	--	H	L	--	--	--	--
Lamprey	Petromyzon marinus	--	--	--	S	--	--	--	--	N	--	S	--	H	Q	--	--	C	G

Fig. S6. Conservation table of the *EPAS1* (HIF-2α) H194R variant (rs570553380) across 100 vertebrate species. Among 100 vertebrate genomes, the H194R variant (rs570553380), resulting in an amino acid change from a histidine (H) to an arginine (R), is only reported in the deep-sea African coelacanth, *Latimeria chalumnae*, a pre-historic lobed finned fish. Lampreys, *Petromyzon marinus*, are the only other species to have an amino acid other than histidine (i.e., asparagine) at this locus. This data was extracted from the Vertebrate Multiz Alignment & Conservation (100 Species) track from UCSC Genome Browser.

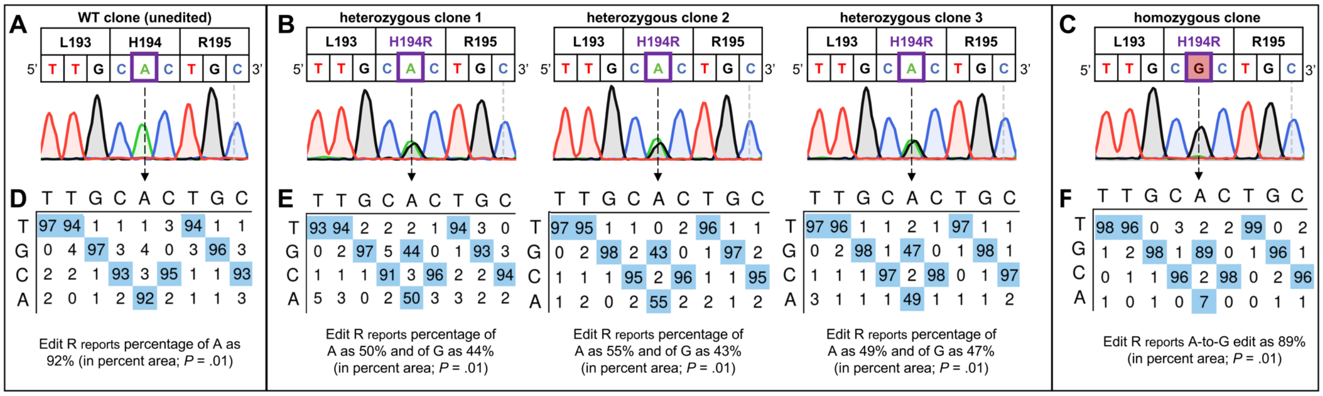

Fig. S7. Sanger sequencing chromatogram of isogenic cells. (A) wildtype (WT, n=1), (B) heterozygous (HET, n=3), and (C) homozygous (HOM, n=1) for the *EPAS1* variant (rs570553380, A>G, p.[His194Arg]) that were transfected, subjected to FACS, and clonally expanded. EditR analysis of the .ab1 files was conducted for the (D) WT clone (92% ‘A’, p = 0.01), (E) HET clones (‘44%, 43% and 49% ‘G’ respectively, p = 0.01), and (F) HOM clone (89% ‘G’, p = 0.01).

A

WTNT

	A	C	G	T
A	0.0%	2.0%	16.1%	1.7%
C	3.1%	0.0%	4.5%	20.9%
G	25.7%	5.1%	0.0%	2.8%
T	0.8%	16.1%	1.1%	0.0%

WTT

	A	C	G	T
A	0.0%	1.9%	15.4%	1.7%
C	3.6%	0.0%	4.1%	21.4%
G	26.3%	5.5%	0.0%	2.7%
T	1.0%	15.4%	1.0%	0.0%

HET1

	A	C	G	T
A	0.0%	2.0%	16.8%	1.8%
C	3.2%	0.0%	4.8%	20.5%
G	25.9%	4.3%	0.0%	2.5%
T	0.7%	16.4%	1.1%	0.0%

HET2

	A	C	G	T
A	0.0%	2.0%	16.4%	2.0%
C	2.9%	0.0%	4.9%	18.9%
G	26.2%	4.3%	0.0%	2.9%
T	1.0%	17.0%	1.4%	0.0%

HET3

	A	C	G	T
A	0.0%	2.0%	16.4%	2.0%
C	2.9%	0.0%	4.9%	19.0%
G	26.2%	4.3%	0.0%	2.9%
T	1.0%	17.0%	1.4%	0.0%

HOM

	A	C	G	T
A	0.0%	1.9%	16.2%	1.5%
C	3.5%	0.0%	4.6%	21.7%
G	25.4%	5.0%	0.0%	2.1%
T	0.8%	16.3%	1.0%	0.0%

B

	WTNT	WTT	HET1	HET2	HET3	HOM
Relative Mutation Rate (A•T to G•C)	1.00	0.96	1.03	1.04	1.04	1.01

Fig. S8. Evaluation of isogenic HEK293T clones wildtype, heterozygous, and homozygous for rs570553380 following whole-exome sequencing. (A) Matrix of single nucleotide variant (SNV) mutation rates observed in exome sequencing data generated from each single-cell expanded population (wildtype-transfected, WTT, heterozygote 1-3, HET1-3, homozygote, HOM) and wildtype non-transfected control (WTNT). (B) Whole-exome A•T to G•C mutation rate of transfected cell lines (WTT, HET1-3, HOM) relative to that of the non-transfected control (WTNT).

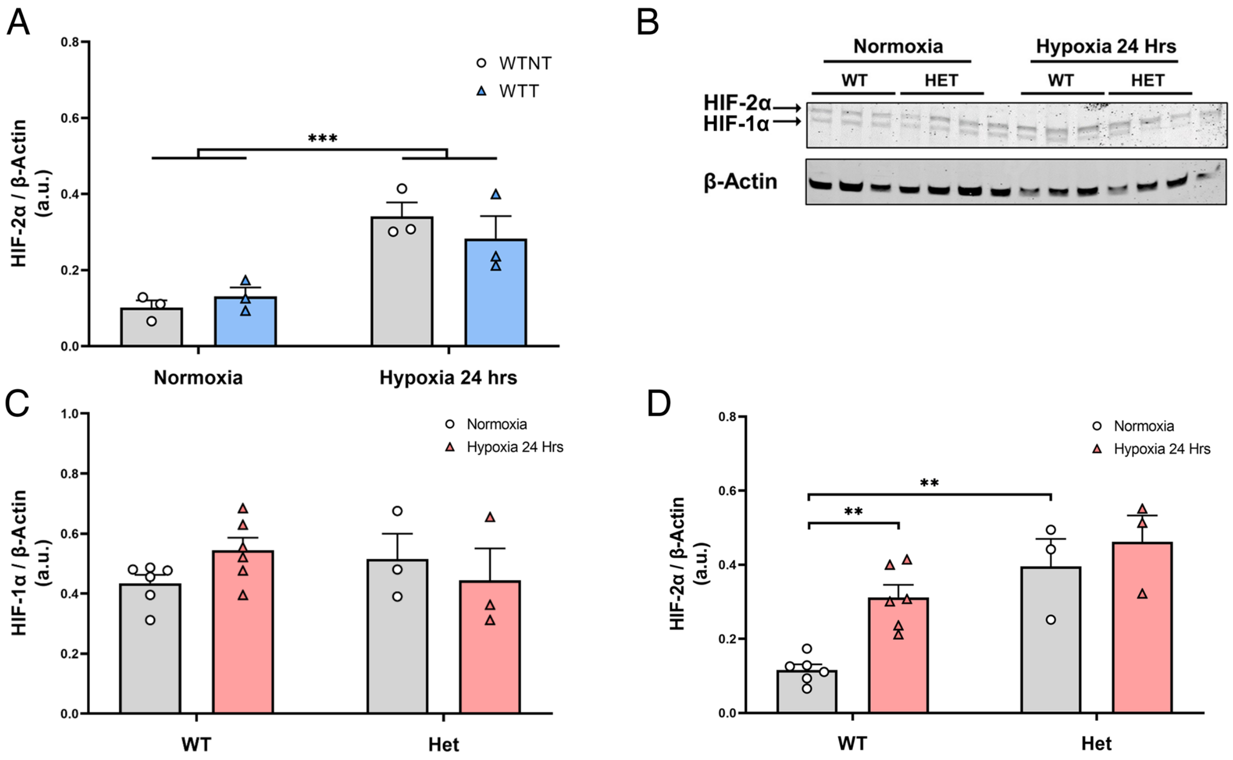

Fig. S9. HIF-2α protein levels are elevated in hypoxia and in the presence of rs570553380 (A>G, p.[His194Arg]) allele. (A) Quantification of Western blot assays performed in HEK293T cells wildtype for SNV rs570553380 revealed a hypoxia effect after 24 hours of relative HIF-2α expression independent of the transfection and clonal expansion processes ($p < 0.001$). Both wildtype cells that had (wildtype transfected, WTT) and had not (wildtype non-transfected, WTNT) undergone transfection and clonal expansion processes were used as controls and showed no significant differences in HIF-2α levels in neither hypoxia (1% O_2) nor normoxia (21% O_2). (B) Western blot analyses of HEK293T cells wildtype (WTT + WTNT) and heterozygous (HET) for rs570553380 (A>G) showed (C) no hypoxia or genotype effects on HIF-1α levels. However, (D) a hypoxia and genotype effect on HIF-2α levels ($p < 0.01$, $n_{WT} = 6$, $n_{Het} = 3$) was observed, in which wildtype cells exhibited lower levels of HIF-2α than cells heterozygous for SNV rs570553380 in normoxia, suggesting potential compensatory changes in response to altered HIF activity.

Table S1. Age estimation of SNVs rs570553380, rs7559484, and rs7556828 in 1000 Genomes Project. Genealogical Estimation of Variant Age (*GEVA*) predicted the ages of rs570553380 (A>G), rs7559484 (T>C), and rs7556828 (G>A) as approximately 9,808, 1,135,393, and 1,312,778 years, respectively. In a separate analysis, the age of rs570553380 was additionally calculated within the 40 Andean whole genomes to be 13,027 years old, in which the derived allele ('G') occurs on the selected haplotype. The generation length for all analyses was set to 25 years.

	Mode	Mean	Median	95% confidence interval	Quality score
Age of rs570553380 (Years)	9,868	9,845	9,808	7,947-11,885	0.955
Age of rs7559484 (Years)	1,135,393	1,135,398	1,134,545	1,065,428-1,207,243	0.824
Age of rs7556828 (Years)	1,312,778	1,312,643	1,311,795	1,232,803-1,392,723	0.836

Table S2: Summary of physiological data and genotype counts in the Andean cohort. The sample size (N), mean and standard deviation of physiological measurements of participants stratified by sex. Continuous measurements are presented as: mean ± SD. Measurements for excessive erythrocytosis (EE), non-EE, hematocrit (%), and O_2 saturation at 10% inspired O_2 are further described by genotype for rs570553380 (A>G) in Andean males and females from this study.

	**N**	**Average (Male)**	**Average (Female)**
Age (Year)	188	41 ± 13	40 ± 13
Height (cm)	188	1.61 ± 0.05	1.49 ± 0.06
Weight (kg)	188	68.5 ± 9.8	63.5 ± 11.4
BMI	188	26.2 ± 3.5	28.7 ± 4.8
EE	60	35%	10%
non-EE	128	0.65	0.90
Hematocrit (%)	188	60.1 ± 7.4	50.8 ± 6.5
O_2 Saturation at 10% (F_{IO_2})	77	85.9 ± 4.2	87.1 ± 2.6

		Genotype Counts			
	**N**	**AA (Male)**	**AG (Male)**	**AA (Female)**	**AG (Female)**
EE	60	54	0	5	1
non-EE	128	77	8	31	12
Hematocrit (%)	188	131	8	36	13
O_2 Saturation at 10% (F_{IO_2})	77	51	4	14	8

Table S3. ***In silico*** **structural analysis and prediction of rs570553380 (A>G, p.[His194Arg]).** *In silico* analysis of the H194R variant on the structure and stability of HIF-2α (PDB ID: 4ZP4) via (A) PROVEAN, (B) MutPred2, and (C) Dynamut2 predict this variant to be deleterious, destabilizing, and influential in DNA binding.

<table>
<tr><th>Software</th><th>Reference</th><th colspan="3">Prediction</th></tr>
<tr><td>PROVEAN</td><td>(46)</td><td colspan="3">PROVEAN predicts the EPAS1 H194R variant to be deleterious, with a PROVEAN score of -6.976, which is significantly over the threshold (PROVEAN score < -2.5) for deleteriousness prediction.</td></tr>
<tr><td rowspan="8">MutPred2</td><td rowspan="8">(47)</td><td colspan="3">MutPred2 predicts the Eukaryotic Linear Motifs (ELMs) and molecular mechanisms that can be potentially affected due to the EPAS1 H194R mutation.</td></tr>
<tr><td colspan="3">Predicted Affected ELM Accessions</td></tr>
<tr><td colspan="3">ELME000052, ELME000062, ELME000233, ELME000336</td></tr>
<tr><td>Altered Molecular Mechanisms Predicted</td><td>Probability</td><td>P-value</td></tr>
<tr><td>Altered metal binding</td><td>0.47</td><td>0.00091</td></tr>
<tr><td>Altered Disordered interface</td><td>0.41</td><td>0.0031</td></tr>
<tr><td>Gain of strand</td><td>0.26</td><td>0.05</td></tr>
<tr><td>Altered DNA binding</td><td>0.21</td><td>0.02</td></tr>
<tr><td>Dynamut2</td><td>(48)</td><td colspan="3">Dynamut2 predicts the change in stability introduced by the mutation. EPAS1 H194R is predicted to be destabilizing.</td></tr>
</table>

Table S4. Variants identified between edited and unedited HEK293T cell lines through whole-exome sequencing. Wildtype-transfected (WTT), wildtype non-transfected (WTNT), heterozygote (HET1, HET2, HET3), and homozygote (HOM) cell lines were submitted for whole-exome sequencing to assess potential off target mutations and the genetic background between clones. Variants were additionally evaluated for occurrence in hypoxia pathway genes within the Gene Ontology term "Cellular Response to Hypoxia" (GO:0071456), in which none were enriched. (Attached as Excel file).

Table S5. Canonical HIF-2α target genes and corresponding Taqman gene expression assay IDs. The gene targets used for Quantitative real-time PCR were assayed in prior studies assessing HIF-2 activity.

Gene	Gene symbol	Assay ID
Actin Beta	*ACTB*	Hs03023943_g1
Adrenomedullin	*ADM*	Hs00181605_m1
Basic Helix-loop-helix Family Member e40	*BHLE40*	Hs00186419_m1
Solute Carrier Family 2 Member 1	*GLUT1 (SLC2A1)*	Hs00892681_m1
Hypoxia Inducible Lipid Droplet Associated	*HLPDA*	Hs00203383_m1
Inhibitor of Growth Family Member 4	*ING4*	Hs01088026_m1
Vascular Endothelial Growth Factor A	*VEGF*	Hs00900055_m1

Table S6. Known and unknown metabolites associated with hematocrit in Andeans and within the FINRISK cohort. (Attached as Excel file).
